# Extralevator abdominoperineal excision (ELAPE) for rectal cancer—short-term results from the Swedish Colorectal Cancer Registry. Selective use of ELAPE warranted

**DOI:** 10.1007/s00384-014-1932-9

**Published:** 2014-06-21

**Authors:** Mattias Prytz, Eva Angenete, Jan Ekelund, Eva Haglind

**Affiliations:** 1 Department of Surgery, NU Hospital Group, Trollhättan, Sweden; 2 Department of Surgery, Sahlgrenska University Hospital/Östra, Gothenburg, Sweden; 3 Scandinavian Surgical Outcomes Research Group (SSORG) and the Sahlgrenska Academy, University of Gothenburg, Gothenburg, Sweden

**Keywords:** APE, ELAPE, Colorectal cancer

## Abstract

**Purpose:**

Local recurrences are more common after abdominoperineal excision (APE) than after anterior resection of rectal cancer. Extralevator APE was introduced to address this problem. This prospective registry-based population study aims to investigate the efficacy of extralevator APE (ELAPE) in improving short-term oncological outcome.

**Methods:**

All Swedish patients operated with any kind of abdominoperineal excision and registered in the Swedish Rectal Cancer Registry 2007–2009 were included (*n* = 1,397) and analyzed with emphasis on the perineal part of the operation. Short-term perioperative and oncological results were collected from the registry.

**Results:**

Extralevator APE did not result in fewer intraoperative perforations or involved circumferential resection margins as compared to standard APE for the entire group. Intraoperative perforations were significantly fewer for patients with low tumours (≤4 cm) (ELAPE: *n* = 28/386 versus APE: *n* = 9/58) (*p* = 0.043) and for early (T0–T2) T-stages (ELAPE: *n* = 3/172 versus APE: *n* = 6/75) (*p* = 0.025). There were significantly more post-operative wound infections for ELAPE than for APE (*n* = 106 (20.4 %) versus *n* = 25 (12.0 %), *p* = 0.011).

**Conclusions:**

The short-term results indicate that selective use of extralevator APE can be warranted, for example, for subgroups with low tumours. In conclusion, selective use of the extralevator APE is advocated as not all patients seem to benefit from the technique, and there are significantly more short-term complications after extralevator APE.

## Introduction

**Fig. 1:**
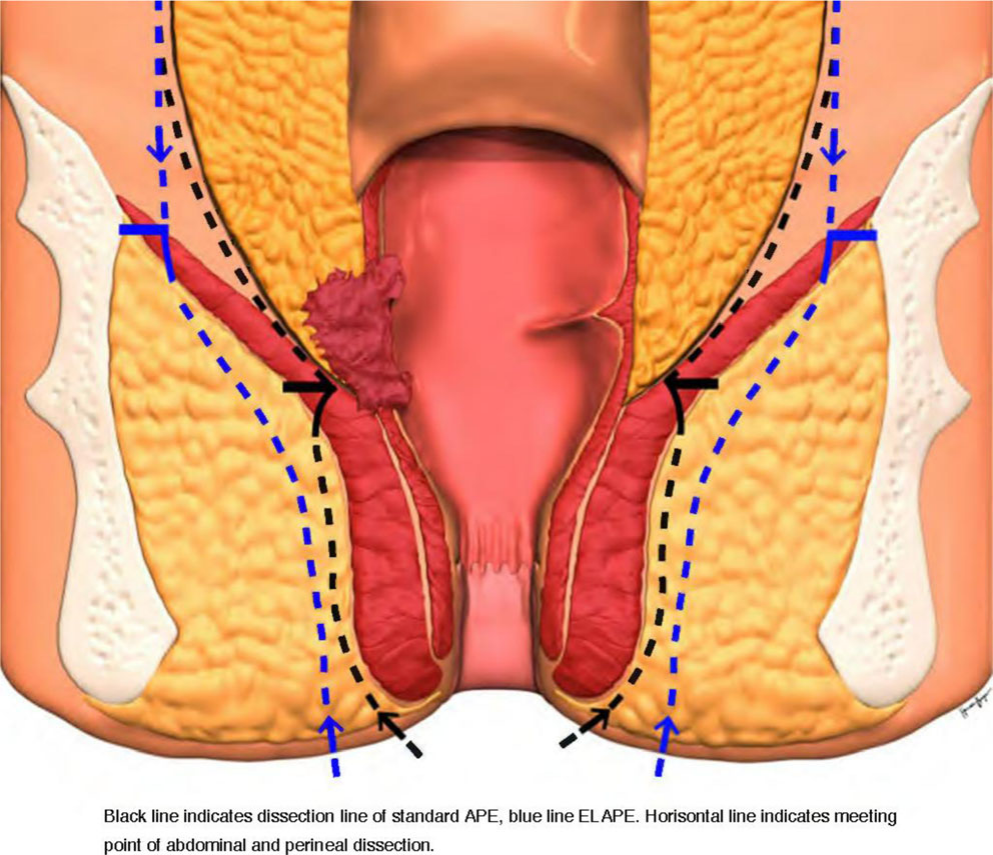
Schematic anatomic picture of dissection in standard APE, as depicted by the *black interrupted line*, and extralevator APE (ELAPE) as depicted by the *blue interrupted line*

Abdominoperineal excision (APE) is the surgical treatment for patients with distal rectal cancer in whom an anterior resection with anastomosis (AR) cannot be performed [1, 2]. Studies show that the overall prognosis for these patients is worse than that for patients undergoing AR and that the local recurrence rates are higher [3-9]. In order to address this problem, a more extensive surgical procedure has been proposed [10-14]. The aim of the procedure—described elsewhere and here referred to as the extralevator APE (ELAPE) ]—is to create a cylindrical specimen without a ‘waist’ in order to minimize the risk of a tumour involvement of the circumferential resection margin (CRM) and to reduce the risk of intraoperative tumour perforation (Fig. 1). Several studies have reported that ELAPE is superior to standard APE [10, 11, 15-17], but others have reported conflicting results [18, 19], and two systematic reviews [20, 21] came to different conclusions as to what extent ELAPE is oncologically superior compared to standard APE. There is one randomized, controlled trial [22] that has reported results in favour for ELAPE regarding local recurrence.

This study aims to evaluate the short-term perioperative and surrogate oncological results in a population-based cohort from the Swedish Colorectal Cancer Registry (SCRCR) following standard APE and extralevator APE.

## Method

A detailed description of the study has previously been published [23].

### Data from the Swedish Colorectal Cancer Registry

The Swedish Colorectal Cancer Registry was established in 1995 for the registration of details regarding the treatment of rectal cancer in Sweden. All Swedish hospitals report to the registry [24]. All perioperative data in the registry on patients operated between 2007 and 2009 were retrieved including cTNM classification; level of tumour from the anal verge; patient demographics; ASA classification; pre- and post-operative adjuvant treatment; certain aspects of the operative technique such as open or laparoscopic operation, level of vascular division and perioperative complications including perioperative bleeding, perforation of the specimen and operating theatre time. Post-operative data such as pTNM-classification, CRM, distal margin, lymph node harvest and involvement were retrieved as were post-operative complications including infections, re-operations and death within 30 days.

Not included in the registry are details on the type of perineal dissection performed. Operative notes for each patient were retrieved from the hospital where the patient was operated. A clinical record form (CRF) was developed and used to register details of the perineal part of the procedure such as position of the patient, division of the levator muscle, removal of the coccyx and the perineal reconstruction. Using this information, the patients were classified as belonging to one of the following: ‘APE’, operated with a traditional APE, ‘ELAPE’, operated with an extralevator APE, ‘Not stated’, where the type of perineal dissection was not possible to be classified from the operating notes.

### Statistics

All data were collected in a database, and statistical analysis was performed using SPSS 21.0 (IBM SPSS Inc. Armonk, NY, USA) and SAS v.9 (SAS Institute). Continuous outcome variables were compared between the groups APE and ELAPE using Wilcoxon’s rank-sum test; categorical data were compared between the two groups using Fisher’s exact test or the chi-squared test, as deemed appropriate.

## Results

**Fig. 2:**
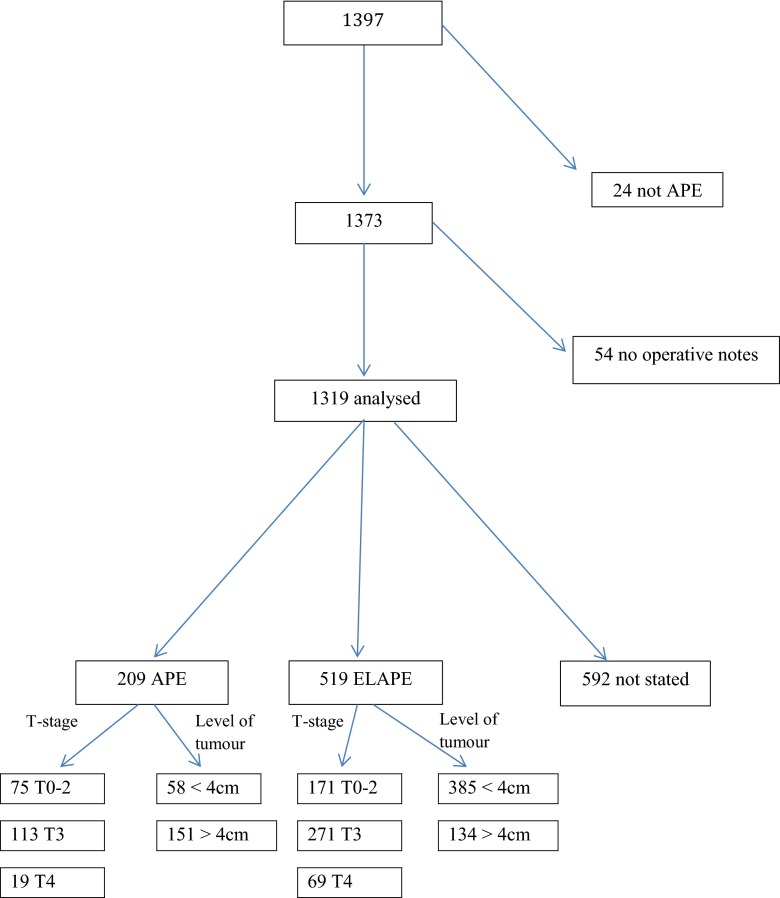
Consort diagram

The cohort consisting of all patients registered as operated due to rectal cancer with abdominoperineal excision during 2007–2009 was identified in the SCRCR. This resulted in a total of 1,397 patients. In the flow chart (Fig. 2), exclusions are described, leaving 1,319 patients for the analysis. For the group excluded due to lack of surgical notes (4 %), clinical and demographic data from the registry did not differ compared with the patients included in the study. Clinical and demographic data for the patients are summarized in Table 1. There was a difference in pT-stage between the three groups but no difference between APE and ELAPE. Outcomes for the ‘not stated’ group were not analyzed further, as no information regarding the perineal part of the APE could be obtained.

**Table 1:** Patient demographics and tumour characteristics in abdominoperineal excision (APE), extralevator abdominoperineal excision (ELAPE) and the group without description of the type of perineal dissection (‘not stated’)

	APE	ELAPE	‘Not stated’	Total	*p* value
Number of patients	209 (15.8)	518 (39.3)	592 (44.5)	1319	
Age, years (median and quartiles 1 and 3)	71 (63–79)	68 (61–76)	70 (62–78)	69 (62–77)	0.005
BMI, kilogram per metre squared (median and quartiles 1 and 3)	25.1 (22.3–27.9)	25.0 (22.6–28.1)	24.9 (22.8–27.7)	25.0 (22.6–27.8)	0.66
Female/Male (%)	93/116 (44/56)	209/309 (40/60)	228/364 /39/61)	530/789 (40/60)	0.30
ASA, 1/2/3/4 (%)	35/113/57/2 (17/55/27/1)	115/281/104/4 (23/56/21/1)	124/320/121/5 (22/56/21/1)	274/714/282/11 (21/56/22/1)	0.06^d^
pT-stage, 0–2/3/4 (%)	75/113/19 (36/55/9)	172/270/69 (34/53/13)	237/314/32 (41/54/5)	484/697/120 (37/54/9)	0.27
pN-stage, 0/1/2/X (%)	121/37/46/5 (58/18/22/2)	288/125/93/12 (56/24/18/2)	324/152/103/12 (55/26/17/2)	733/314/242/29 (56/24/18/2)	0.12^e^
M-stage, 0/1/X (%)	170/35/4 (81/17/2)	461/44/12 (89/9/2)	512/58/18 (87/10/3)	1143/137/34 (87/10/3)	0.001^f^
Tumour differentiation, low/medium/high/unknown (%)	27/151/16/14 (13/78/8/7)	75/358/33/52 (14/69/6/10)	102/391/42/51 (17/67/7/9)	204/901/91/117 (16/69/7/9) 0.71	0.71
Mean tumour height^a^	6.6	3.4	4.1	4.2	<0.001
Tumour height <2 cm	17 (8)	176 (34)	157 (27)	350 (27)	
Tumour height 2–4 cm	41 (20)	210 (41)	198 (34)	449 (34)	
Tumour height 4–5 cm	36 (17)	62 (12)	101 (17)	199 (15)	
Tumour height >5 cm	115 (55)	66 (13)	125 (22)	306 (23)	
Pre-operative radiotherapy, *n* (%)	144 (69)	456 (88)	472 (80)	1072 (81)	<0.001
Pre-operative radiochemotherapy, *n* (%)	32 (15)	159 (31)	88 (15)	279 (21)	<0.001
Type of radiotherapy, short^b^/long^c^/other	109/34/1 (76/24/1)	284/166/5 (62/36/1)	365/99/5 (78/21/1)	758/299/11 (71/28/1)	0.004

Percentages are given in parenthesis

^a^Distance in centimetre from the lower edge of the tumour to the anal verge

^b^Short radiotherapy equals 5 Gy × 5

^c^Long radiotherapy equals 1.8/2.0 Gy × 25

^d^For the analysis ASA3 and ASA4 were grouped together

^e^pNx were excluded in the analysis

^f^MX were excluded in the analysis

Operative data on the two groups APE and ELAPE are summarized in Table 2 and show significant differences in patient positioning, resection of the coccyx, type of perineal reconstruction, type of technique used for the abdominal part of the procedure between the groups and a longer operation time for ELAPE.

**Table 2:** Operative data for abdominoperineal excision (APE) and extralevator abdominoperineal excision (ELAPE)

Variable	APE	ELAPE	*p* value
Lithotomy/Jackknife^a^ (%)	138/69 (66/33)	106/410 (21/79)	<0.001
Coccyx resection (%)	12(6)	248 (48)	<0.001
Reconstruction: suture/mesh/flap^b^ (%)	207/1/0 (99/1/0)	265/123/125 (51/24/24)	<0.001
Open/Laparoscopic (%)	204/5 (98/2)	482/35 (93/7)	<0.02
Operating theatre time, minutes (mean (SD))	339 (116)	380 (151)	<0.001
Perioperative bleeding, millilitre (mean (SD))	884 (995)	793 (831)	0.43

Reporting patient position, resection of the coccyx, type of perineal reconstruction, surgical technique for the abdominal operation, operating room time and perioperative bleeding

^a^Refers to patient positioning during the perineal part of operation

^b^Mesh refers to any kind of absorbable/non-absorbable mesh, and flap refers to any kind of myocutaneous flap

Comparisons of APE and ELAPE groups showed no differences regarding the various details of the oncological result from the pathology report. Complications did not differ except for wound infections, significantly more often found in the ELAPE group (Table 3). In the analysis of subgroups, we selected tumours below and up to 4 cm from the anal verge and pT stages (0–2, 3 and 4, respectively). There were significantly lower rates of perforations in the ELAPE for tumours below 4 cm and for pT0–2, respectively, (Table 4). Regarding pT3 or pT4, there were no differences between the techniques.

**Table 3:** Oncological data as found in the pathology report and complications in APE and ELAPE groups

Variable	APE	ELAPE	Missing^a^	*p* value
Bowel perforation (%)	23(11)	40 (8)	3	0.19
Involved CRM (%)	13(6)	53 (10)	1	0.12
CRM in millimetres (median (quartiles 1 and 3))	7 (3–13)	6 (3–10)	110	0.052
Number of lymph node harvested, mean (SD)	15.7 (9.7)	13.7 (8.5)	1	0.13
30-day mortality (%)	5(2.4)	11(2.1)	14	0.79
Complications, all (%)	87 (41.6)	238 (45.9)	0	0.32
Wound infections^b^ (%)	25 (12)	106 (20.5)	^c^	0.008
Re-operations (%)	19 (9.1)	61 (11.8)	1	0.36

^a^Missing refers to missing data, number of

^b^Wound infections includes all wound infections, abdominal and perineal wounds

^c^Only those with infections are reported in the registry

**Table 4:** Oncological data for tumour height with the lower limit ≤4 cm from the anal verge and by T-stage for APE and ELAPE groups

Subgroup	APE	ELAPE	Missing^a^	*p* value
Tumour height ≤4 cm	58	386		
Perforation, yes/no, (%)	9/49 (16/84)	28/357 (7/93)	0/1	0.043
Involved CRM, yes + uncertain/no (%)	4/54 (7/93)	36/350 (9/91)	0/0	0.81
CRM in millimetres, median/mean (quartiles 1 and 3)	5/7.3 (3–10)	6/7.8 (3–10)	8/57	0.87
T 0–2	75	172		
Perforation, yes/no (%)	6/69 (8/92)	3/168 (2/98)	0/1	0.025
Involved CRM, yes + uncertain/no, *n* (%)	0/75 (0/100)	3/169 (2/98)	0/0	0.56
CRM in millimetres, median/mean (quartiles 1 and 3)	10/11.4(5–13)	10/11.0 (5–15)	20/42	0.58
T 3	113	270		
Perforation, yes/no (%)	15/98 (13/87)	20/248 (7/93)	0/2	0.082
Involved CRM, yes + uncertain/no (%)	9/104 (8/92)	23/246 (9/91)	0/1	
CRM in millimetres, median/mean (quartiles 1 and 3)	7/9.4 (2–12)	5/6.6 (3–10)	6/22	0.069
T 4	19	69		
Perforation, yes/no (%)	2/17 (11/89)	17/52 (25/75)	0/0	0.23
Involved CRM, yes + uncertain/no (%)	4/15 (21/79)	27/42 (39/61)	0/0	0.18
CRM in millimetres, median/mean (quartiles 1 and 3)	3.5/9.0 (1.1–11)	1.5/4.4 (0–6.5)	3/12	0.13

*n* Denotes numbers throughout

^a^Missing refers to the number of missing data

## Discussion

In this national study of 1,319 patients with abdominoperineal excision for rectal cancer, there was no oncological advantage using ELAPE as compared to APE for the entire group with regard to engaged CRM and intraoperative bowel perforation. However, in subgroups consisting of the most distal tumours or with tumours staged pT0–T2, there were significantly fewer perforations in the ELAPE group. The results of the study support previous findings that ELAPE can reduce the risk of intraoperative bowel perforation, for the most distally situated tumours.

West et al. [10, 11] reported a lower perforation rate and fewer cases with involved CRM using ELAPE in case series in comparison with historical controls that had high rates of both perforations and involved margins. Stelzner et al. [20] concluded from their systematic review of 14 non-randomized studies from 1997 to 2011 on ‘extended APE’ and 50 studies on traditional APE from 1991 to 2011 that ‘extended APE’ had a reduced risk of intraoperative perforation. The effects on local recurrence and survival rates were not possible to be analyzed. Others have been unable to confirm these findings in case series with historical controls [18, 21], and Krishna et al. [21] concluded in their comparison of published rates of CRM involvement and intraoperative bowel perforations that there was no convincing evidence that ELAPE results were better than those for standard APE.

The only randomized controlled study by Han et al. reported a reduced recurrence rate following ELAPE after median follow-up of 29 months, suggesting that there is an oncological advantage with ELAPE as compared to traditional APE in patients with T3 and T4 tumours [22]. However, their study was small (*n* = 67), no details of external or internal validity were reported, and less than 30 % of the patients received neoadjuvant treatment. Due to these methodological weaknesses, their finding may be regarded as interesting but not as conclusive.

We confirm previous findings of a higher rate of post-operative infectious complications in the ELAPE group as compared to standard APE [11, 18, 19, 22], but we found no differences between the groups regarding reoperation rate, overall complication rate or short-term mortality. Data on wound-related infections were retrieved from the national registry, and the rates that we found were not in the higher ranges among the reported. We have, however, no reason to suspect that there was any bias in reporting complications to the Swedish Colorectal Cancer Registry between the two types of procedures, and the registry did not differentiate between perineal and laparotomy wound infections.

A majority of the patients received neoadjuvant treatment, and this was more common among the patients in the ELAPE group, which could influence long-term results. Whether this, to some extent, can explain the increased wound infection rate is not clear.

A strength of our study is the cohort size and the fact that the patients included comprises 94 % of all Swedish patients operated with any kind of APE in the years 2007–2009. Thus, the results are population-based. Selection bias is not a problem since practically every Swedish patient in this time frame was included. This is a strength compared to other studies with more selected case series. The Swedish Colorectal Cancer Registry is well established, has a high coverage and is validated and updated [24-26]. The cohort is prospectively registered, and the time frame of the study is short and recent. There are no comparisons to historical data.

There are, however, some weaknesses in the study. Although the coverage is high, there are still missing data. For most variables reported, the rate of missing data is small and ranges from 0 to 3 % for the variables used in our study with the exception of the numerical value (in mm) for the CRM, with missing data in 20 % of cases, whereas the data on involvement of the CRM and intraoperative perforation were missing in less than 1 %. Our results regarding the absolute value of the CRM must therefore be interpreted with caution. The finding of a tendency towards a wider CRM in millimetres for the traditional APE-group may be due to a larger number of tumours situated higher in the rectum in the traditional APE group that consequently have a wider distance to the CRM—since they are to a larger extent covered by the mesorectum.

In the registry, complications were registered including reoperations, but in the time frame of our study, complications were not graded according to the Clavien-Dindo classification [27, 28], and the complication rates may be underreported [26]. However, the total incidence of complications is similar to other prospective studies of rectal cancer surgery [29]. The registry does not include details of the perineal part of the operation, and therefore, details of this had to be collected separately. Unfortunately, it became evident that Swedish surgeons often did not include details of the perineal dissection in their operative notes, but the remaining sample is still large.

ELAPE was already widely spread among Swedish surgeons in 2007–2009, but it was used with some discrimination for rectal cancers situated low to very low in the rectum. This selective use makes comparisons of outcomes more difficult. This is particularly true for T3 and even more for T4 tumours below 4 cm from the anal verge for which no differences between APE and ELAPE were found. On the other hand, one could argue that the ELAPE group could have included more of the advanced or ugly tumours and that the groups differed as to tumour classification as the registry did not include every detail of classification.

The finding of fewer intraoperative perforations for the early tumours (pT0–pT2) with ELAPE is somewhat unexpected and should be mentioned. A plausible hypothesis is that these perforations to some extent is due to downstaging of tumours from neoadjuvant radiotherapy which renders the tumours more fragile and susceptible to perforations when the dissection is performed in closer vicinity to the tumour—as it is with traditional APE.

Other variables that can be attributed to the results of both standard APE and ELAPE are surgeon-related variables such as level of training and annual numbers of cases. Swedish colorectal surgeons at the time could be considered well trained due to well-attended workshops on TME surgery. However, in the study, there are both high-caseload centres and centres that performed less than 10 procedures annually, so there is definitely a variation in surgeon-related competence. Since the study included all Swedish patients and centres during these years, the results should have external validity on a population basis. Another factor that might influence results is patient positioning during the perineal part of the operation; however, since the prone jackknife position is used in the majority of the ELAPE procedures and the lithotomy position in the majority of the traditional APE procedures, the influence of this factor is not possible to be analyzed.

The rapid implementation of the ELAPE technique in Sweden is interesting particularly in comparison with the implementation of laparoscopic surgery, which in sharp contrast, has not been widely spread in Sweden. At the time (2007–2009), only two small randomized controlled trials (RCTs) had reported results of laparoscopic technique [30, 31], but at present, one large RCT has reported interesting differences regarding in particular the ‘oncologic outcome’ for the low tumours with seeming advantages for the laparoscopic technique [29].

We conclude that selective use of extralevator abdominoperineal excision is advocated based on the short-term results of the implementation of the method in Sweden.
